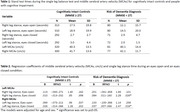# Unravelling the link: complex motor function, cerebral blood flow, and cognitive impairment

**DOI:** 10.1002/alz70856_104082

**Published:** 2025-12-25

**Authors:** Laura Keiko Fitzgibbon‐Collins, Nour Moussa, Solve Elmstahl, Arkadiusz Siennicki‐Lantz

**Affiliations:** ^1^ Western University, London, ON, Canada; ^2^ Lund University, Lund, Scania, Sweden

## Abstract

**Background:**

Subtle alterations of complex motor function, such as balance and gait, are observed in the early onset stages of Alzheimer's Disease. Similarly, reduced cerebral blood flow is associated with the progression and development of dementia. We sought to determine if increased cerebral blood flow is associated with better balance in a population‐based cohort of older adults, and if this relationship differs between people who are cognitively intact and people who are at increased risk of a dementia diagnosis.

**Methods:**

401 participants from a Swedish cohort “Good Aging in Scania” were grouped according to their general cognitive level with a Mini‐Mental State Examination (MMSE, Table 1); whereby a MMSE score of <27, participants were categorized as having an increased risk of dementia and a MMSE score of ≥27 participants were categorized as being a cognitively intact control. Participants completed a bilateral collections of middle cerebral artery velocity (MCAv) as well as an eyes open and an eyes closed single leg balance test (Table 1). Participants were asked to stand as long as possible on a single limb in both the eyes open and eyes closed conditions. A regression analysis was completed in both groups, investigating MCAv (cm/s) and single leg stance time (seconds) for the eyes open and eyes closed conditions. Significance was set to *p* ≤0.05.

**Results:**

After adjustment for age and sex, participants at risk of a dementia diagnosis (MMSE<27), had a significant and positive association between MCAv and the one leg balance test during the eyes closed conditions (right leg stance *p* = 0.026, left leg stance *p* = 0.016, Table 2). No association was observed for the eyes open single leg balance test or among cognitively intact controls (MMSE ≥27) in the eyes open and eyes closed conditions (Table 2).

**Conclusion:**

In people with lower general cognition and a risk for developing dementia or mild cognitive impairment, a better overall cerebral perfusion might compensate for cognitive and motor integration deficits enabling longer standing times. Without visual input, participants might not mask deficits in sensory and motor control, making cerebral perfusion a limiting factor.